# Ethnobotanical investigations and urban utilization potential of medicinal and edible Lamiaceae plants: a case study from Guizhou, China

**DOI:** 10.3389/fphar.2025.1601710

**Published:** 2025-09-03

**Authors:** Mengxue Yang, Huan Wu, Yanfei Geng

**Affiliations:** ^1^ College of Life Sciences, Guizhou University, Guiyang, China; ^2^ College of Tea Science, Guizhou University, Guiyang, China

**Keywords:** Lamiaceae plants, *Elsholtzia rugulosa* tea, medicinal and edible plants, Guizhou, chemical analysis

## Abstract

**Introduction:**

The unique geographical environment of Guizhou Province, China, has nurtured rich resources of Lamiaceae. The Dong people have developed diverse medicinal and edible utilization methods for Lamiaceae plants through long-term practice. This study mainly aims to (1) document the medicinal and edible uses of Lamiaceae plants in the Dong community of Yangwei Village, Shangzhong Town, Liping County, Guizhou; (2) evaluate the chemical composition of five Lamiaceae species; and (3) provide a detailed account and assessment of the tea-making process of *Elsholtzia rugulosa*, a commonly used local plant, while exploring its potential for development as a specialty tea beverage.

**Material and methods:**

Yangwei Village (Liping County), a representative Dong ethnic community with rich ethnobotanical knowledge of medicinal and edible plants, was selected as main study site. Ethnobotanical knowledge of Lamiaceae plants was collected through semi-structured interviews with key informants and participatory observations. Five Lamiaceae plants were chemically analyzed, with *E. rugulosa* selected as the primary research subject due to its traditional applications, rich bioactive compounds, and good processing adaptability. The processing workflow of *E. rugulosa* tea was thoroughly investigated, with improvements based on standard green, oolong, and black tea procedures. The resulting three flavored tea broths were then subjected to sensory and compound evaluations.

**Results:**

The survey documented 101 Lamiaceae species (39 genera), mostly herbs. Chemical analysis indicated significant development potential for *Prunella vulgaris*, *Leonurus japonicus*, and *Perilla frutescens*. Sensory evaluation showed optimized *E. rugulosa* infusions had translucent color, pure aroma, and mellow taste. The black tea flavored infusion exhibited higher total phenolics and flavonoids; the oolong tea flavored infusion excelled in free amino acids and dry matter; and the green tea flavored infusion contained the highest water extract. The optimized *E. rugulosa* tea enhanced taste and preserved bioactive compounds, aligning with the urban demand for health, naturally derived, and culturally meaningful beverage products.

**Conclusion:**

The findings documents Dong-specific ethnobotanical knowledge in Yangwei Village Lamiaceae plants, which is valuable for both conservation and sustainable use. The medicinal plants from the Lamiaceae family have significant development potential, especially in urban use and commercialization.

## 1 Introduction

Since ancient times, the concept of “the same origin of medicine and food” has been deeply rooted (Fang, 2023). The understanding and origins of traditional Chinese medicine (TCM) were initially closely linked to foraging activities. Through countless experiments and observations, ancient people gradually developed the foundational knowledge of pharmacology. Using medicinal and edible plants as raw materials, they created corresponding food products with health benefits for human consumption (Hong et al., 2015).

The Dong people are one of the important minority groups in southern China, possessing unique ethnic culture, lifestyle, and rich traditional ecological knowledge. Plants of the Lamiaceae family, with their widespread distribution, diverse species, and varied bioactive compounds, play an indispensable role in the daily lives of the Dong people. Over the course of long-term adaptation to mountainous environments, the Dong people have accumulated extensive experience in utilizing local plant resources (Zhou et al., 2014), developing diverse methods for the medicinal and edible use of Lamiaceae plants.

The Lamiaceae family is a relatively large plant family with a global distribution range (Ma and Feng, 2017). There are 10 subfamilies in the world, with about 220 genera and more than 3,500 species. Among them, monotypic genera account for about one-third, and oligotypic genera also account for about one-third. There are 99 genera and more than 800 species in China (Wu and Li, 1977). Most of the genera of this family are produced in Asia, Africa and Europe. In recent years, research on Lamiaceae plants has been increasingly in-depth worldwide. Kadioglu et al. (2024) found that Lamiaceae plants occupy an important position among local wild edible plants in their study conducted in the Sivas region of Turkey. Their rich genetic diversity not only supports traditional dietary culture but also possesses irreplaceable ecological conservation value, providing important reference for understanding the cross-regional ecological functions of Lamiaceae plants. At the same time, in their investigation in Ağrı Province, Turkey, they proposed sustainable utilization strategies for wild plants such as Lamiaceae, including regulating collection cycles in line with market demand and establishing a traditional knowledge database, providing a practical model for plant resource management in similar ecological regions (Kadioglu et al., 2020).

Although there have been studies on Lamiaceae plants in terms of classification and medical applications, research on the ethnopharmacology of Lamiaceae plants is still relatively scarce. This study takes Lamiaceae plants in Guizhou Province as its research object and employs ethnobotanical methods, including interviews with key informants, free listing, semi-structured interviews, and literature reviews.

Therefore, the purposes of this study are as follows:

First, to document the utilization patterns of medicinal and edible Lamiaceae plants in Dong communities of Guizhou, Yangwei Village as the primary study site, including local classification and naming systems, life forms, folk collection sites, utilized plant parts, methods of use, and perceived therapeutic or nutritional effects. In addition, the study aims to catalogue and classify the recorded Lamiaceae species and summarize those most commonly used.

Second, to conduct chemical compound analyses on five representative medicinal and edible Lamiaceae species. The analysis will cover key compounds such as dry matter, free amino acids, water-soluble extracts, total phenolics, total flavonoids, rutin, and chlorogenic acid, thereby providing a comprehensive scientific basis for further research and facilitating the effective utilization and value-added development of these plant resources.

Finally, to investigate and record the tea-processing methods of *Elsholtzia rugulosa* in urban contexts, including the preparation of teas with varying flavors, and to evaluate the sensory and chemical characteristics of the resulting tea infusions after steeping.

## 2 Materials and methods

### 2.1 Field survey

**FIGURE 1:**
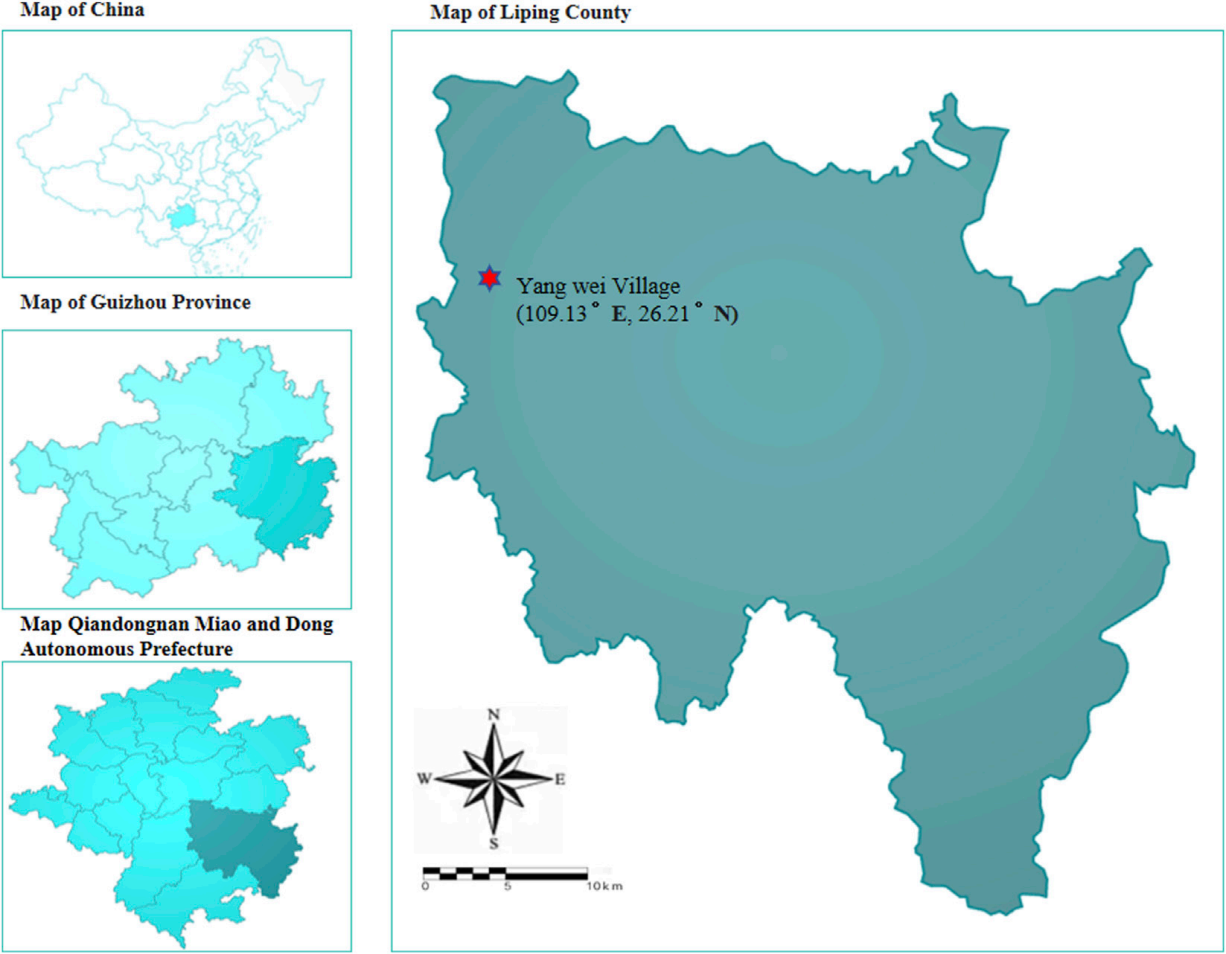
Map of study area.

We conducted field visits to Qianxinan Prefecture, Zunyi City, and Qiandongnan Prefecture, and ultimately selected Liping County for detailed investigation, given its significance as a culturally rich Dong ethnic enclave (Figure 1). Liping County lies in the southeastern part of Guizhou Province and the southern part of Qiandongnan Miao and Dong Autonomous Prefecture. Its geographical coordinates range from 25°41′ to 26°08′ N and from 108°31′ to 109°31′ E (Geographical Environment, 2024). It is 94 km wide from east to west and 112 km long from north to south, with a total area of 4,441 km^2^ (Liping Yearbook Compilation Committee, 2017). Liping is located on the southeastern edge of the slope of the Yunnan-Guizhou Plateau, with the Miaoling Mountains running through the whole area. Its northeastern part borders on the Wuling Mountains. The terrain is high in the northwest and low in the northeast, southeast and southwest (Liping Yearbook Compilation Committee, 2017). Among them, Yangwei Village in Liping County is the main research area. Yangwei Village is located in the northwestern part of Liping County, Qiandongnan Miao and Dong Autonomous Prefecture, and is affiliated with Shangzhong Town. The Dong population in Yangwei Village accounts for 95% of the total population, making it an important settlement of the Dong people. Liping County, characterized by its unique terrain and climate, is abundant in medicinal and edible plant resources, which the Dong people have skillfully utilized in their daily lives.

### 2.2 Ethnobotanical data collection and analysis

We conducted our research using relevant ethnobotanical theories and field survey methods. We developed a standardized semi-structured interview outline to interview key informants (herbalist) to obtain relevant ethnobotanical information. The core content of the interview outline is as follows:

1. Basic information about respondents: age, gender, education level, and whether they engage in herbal-related activities.
2. Plant knowledge:

1. What are the commonly used plants in the local area? (List freely)

2. What are the Dong language names, Chinese names of these plants?

3. Which parts of these plants are utilized?

4. What are the primary purposes for which these plants are used? What are the specific methods of utilization?

5. In what season and at what locations are these plants typically collected?

6. How was this knowledge acquired?

7. For plants with both medicinal and edible uses: Besides their medicinal value, how are they consumed in daily diets?

8. Specific steps and details of the processing methods for particular plants.

The interviews with key informants focused on in-depth medicinal knowledge, including diagnostic approaches for diseases, specific usage methods, efficacy assessment, and the transmission of such knowledge. We particularly focused on their detailed explanations of disease classification, the efficacy of plants, and their treatment experiences. Interviews with other villagers focused on commonly applied knowledge in daily life, such as common methods of consumption, simple home remedies, collection locations and seasons, and basic identification characteristics of plants. The questions were designed to document traditionally shared utilization patterns.

After the interviews, plant specimens were collected and photographed under the guidance of local residents. The collected Lamiaceae plant specimens were identified and cataloged with reference to Flora of China (Editorial Committee of the Flora of China, 1993), and Flora of Guizhou (Editorial Committee of Flora of Guizhou, 2004). All voucher specimens were deposited in the Herbarium of Guizhou University. Additionally, the processing methods of the local tea-like plant *E. rugulosa* were documented through participatory observation with photographic documentation.

The informants were selected by random sampling combined with snowball sampling. A total of 53 local residents from Yangwei Village, Shangzhong Town were interviewed, including 1 village head, 2 village herbalists and 50 farmers. Most of the people interviewed this time were over 60 years old. Most of them had not received formal education and made a living mainly by farming. Their medical knowledge was mainly inherited through self-study or passed down from generation to generation within the family. In the long run, as the older generation of village herbalists passed away and young people, longing for urban life, were unwilling to stay in the village to continue this medical tradition, the usage, application, key techniques and experience related to Dong ethnic medicine would gradually be lost, which would be a significant loss for traditional Chinese medicine.

Refer to the classification system for traditional Chinese medicine in the China Encyclopedia Database (Encyclopedia of China, 2021). Based on the treatment symptoms described by the respondents, the reported Lamiaceae plants were classified according to their TCM effects.

This study strictly adheres to the ethical principles of ethnobotanical research (International Society of Ethnobiology, 2006). Prior to data collection, approval was obtained from the administrative departments of the study area and local village committees. Before each interview, researchers explained the nature and purpose of the study in detail to each potential participant. They emphasized that participation was entirely voluntary, that participants had the right to withdraw from the study at any time without providing a reason, and that they would not suffer any adverse consequences as a result. At the same time, they clearly stated that the content of the interviews would be recorded, that the information collected would be kept strictly confidential, and that it would be used solely for research purposes.

### 2.3 Chemical analysis

In this study, five species from the Lamiaceae family—*Meehania fargesii*, *Prunella vulgaris*, *Leonurus japonicus*, *Perilla frutescens*, and *E. rugulosa*—were selected for chemical analysis. The selection was based on several key considerations: according to preliminary ethnobotanical investigations, these species are widely used within Dong communities and serve as important local resources for both medicinal and dietary purposes. They represent diverse genera and encompass the primary uses of Lamiaceae plants in Dong ethnobotany, thus offering a representative overview of the group. Despite their significance in traditional medicine and food culture, systematic chemical profiling of these plants remains limited. Therefore, analyzing their key bioactive constituents enables a scientific evaluation of their development potential and application prospects.

Chemical analyses were conducted on the aerial parts of all five species. The following components were quantified: dry matter and moisture content (National Health Commission, 2016a), water extract content (National Tea Standardization Technical Committee, 2013), free amino acids (National Health Commission, 2016b), total flavonoids (Zhang, 2020), total phenolics (Ying, 2013), as well as the specific compounds rutin and chlorogenic acid (Wang et al., 2017). These measurements provide a comprehensive profile of the chemical composition of the selected Lamiaceae plants.

To scientifically assess the significance of differences in compound content among the five plant species, all data were analyzed using one-way analysis of variance. If significant differences were found between groups (*P* < 0.05), Tukey HSD test was further used for multiple comparisons. All statistical analyses were performed using SPSS 26.0 software. The results are presented as mean ± standard deviation, and significant differences are indicated using letter codes (different letters in the same column indicate significant differences, *P* < 0.05). Additionally, to comprehensively assess their development potential, this study compared the content of key bioactive compounds with the active compound content of Lamiaceae plants reported in published literature.

### 2.4 Processing and sensory evaluation of *Elsholtzia rugulosa* tea

During our field visits to Liping County, *E. rugulosa* emerged as a standout species widely used by local villagers to prepare herbal teas, reflecting its deep-rooted presence in traditional ethnobotanical knowledge. Unlike many other Lamiaceae plants, this species is not only culturally significant but also functionally versatile. It is rich in bioactive compounds—including volatile oils, flavonoids, polysaccharides, and trace elements—that are associated with a wide range of pharmacological properties, such as antibacterial, antiviral, antioxidant, anti-Alzheimer’s, and hypoglycemic effects (Liu et al., 2012). These health-promoting qualities make *E. rugulosa* particularly well-suited for development as a functional tea, especially in the context of rising health awareness in urban markets.

Local residents traditionally process *E. rugulosa* by sun-drying its leaves for household use, emphasizing its accessibility and everyday health value. However, with modern tea consumers increasingly seeking products that combine health benefits with refined flavor profiles and aesthetic experience, we selected this species for further development. Drawing on traditional methods and inspired by established green, black, and oolong tea processing techniques, we optimized the processing protocols to enhance flavor, texture, and market appeal while preserving the plant’s medicinal qualities.

*Elsholtzia rugulosa* green tea, black tea, and oolong tea were prepared following the standard processing methods for green, black, and oolong teas, respectively (Xu et al., 2018). The processing of *E. rugulosa* green tea involves withering, high-temperature enzyme inactivation, rolling, and drying. A brief heat treatment at 200°C for 3–5 min effectively deactivates enzymes, thereby preserving the original green color and fresh flavor. In contrast, the black tea processing includes withering, rolling, fermentation, and drying, with a prolonged fermentation period (8–10 h) that facilitates oxidative polymerization. The oolong tea follows a similar procedure to black tea but employs a shorter fermentation time (4–5 h), which contributes to the development of a distinctive fruity aroma.

The appearance of tea liquid, aroma, taste and tea leaves quality characteristics of *E. rugulosa* tea were evaluated according to the national standard GB/T 23776-2018 (National Technical Committee for Tea Standardization, 2018), GB/T 14487-2017 (National Technical Committee for Tea Standardization, 2017) and “Tea sensory evaluation Terms” —T/CTSS 58-2022 (Chinese Society of Tea, 2022).

## 3 Results and discussions

### 3.1 Medicinal and edible Lamiaceae plants in the research area

This study investigated a total of 101 species of Lamiaceae plants belonging to 39 genera from 53 local residents, accounting for 39.4% of the total number of genera of Lamiaceae plants in China. Among them, the genus *Salvia* had the largest number of plant species, with 11 species. There were 2 monotypic genera, namely *Heterolamium debile* var. *cardiophyllum* and *Perilla frutescens*. These 101 kinds of plants have been sorted out and catalogued (Supplementary Table S1).

#### 3.1.1 Classification and naming of Lamiaceae plants of the Dong ethnic group

Folk classification refers to the way that people take the morphology, habits and uses of plants as the basis for classification, without considering the genetic and evolutionary relationships among plants, and give plants common names (Wang et al., 2019). The characteristic of folk classification is that it starts from human needs and practicality, is easy to understand, simple and practical, and is convenient for guiding production. The natural classification method determines the genetic relationships and systematic arrangement of plants according to the similarity of plant morphology (Zhang, 1999). The commonly used method at present is the comparative morphological classification method, that is, distinguishing plants by comparing the morphological characteristics of various plant organs, which can objectively reflect the genetic relationships and evolutionary development in the plant kingdom.

The plant classification method of the Dong ethnic group belongs to the artificial classification method, which has strong ethnic cultural characteristics and certain practical value (Yang et al., 2013). The Dong ethnic group classifies the plants that can be eaten by people and livestock as the “vegetable” category, collectively refers to woody plants as “trees”, and calls vines and creeping plants “vines”, and classifies the plants with soft stems among higher plants as the “grass” category. The plants investigated this time are mainly divided into three categories: “Ma” (vegetable), referring to the plants that can be eaten by people and animals; “Nao” (vegetable), referring to the edible vegetables with special odors that can be used as spices; “Nyangt” (grass), usually referring to the plants with soft stems among higher plants except for trees, crops and vegetables, and these plants are generally not eaten by people and are usually used to feed livestock.

The naming of plants of the Dong ethnic group is deeply influenced by the ethnic culture, showing the characteristics of being straightforward and easy to understand, and also has distinct ethnic characteristics and obvious regularity. The Dong ethnic group names plants according to the morphological characteristics, colors, growth habits and growth environments of plants, which is quite similar to the composition of the Linnaean binomial nomenclature. The naming of plants of the Dong ethnic group also consists of several parts. The first word is usually the name of the plant category, the name of the utilized part of the plant or the central noun modified and limited, which is the main part of plant naming. The second and third words behind are usually adjectives that modify and limit the main noun. For example, plants are named in the way of “category word + morphological feature + function”, like *L. japonicus* (Ma cun pang). Here, “Ma” means “vegetable”, “Cun” means “rhizome”, and “Pang” means “nourishing blood”, indicating that *L. japonicus* is a kind of vegetable whose rhizome can be eaten and has the effect of nourishing blood. There is also the way of naming in the form of “Dong ethnic group’s species name word + color feature”, such as *Perilla frutescens* (Nao ya). “Nao” is “fish-flavored coriander” (species name word), and “Ya” is “red”, meaning that *Perilla frutescens* is the red fish-flavored coriander. It can be seen that the names of plants of the Dong ethnic group can vividly describe the basic characteristics of plants.

#### 3.1.2 Life forms, utilized parts, collection locations and usage methods of Lamiaceae plants

The results of the analysis on the life forms of medicinal and edible plants showed that the investigated Lamiaceae plants had two life forms in total, namely herbs and shrubs. Herbs had the highest proportion, with 92 species (91.1%), while there were 9 species of shrubs (8.9%).

**FIGURE 2:**
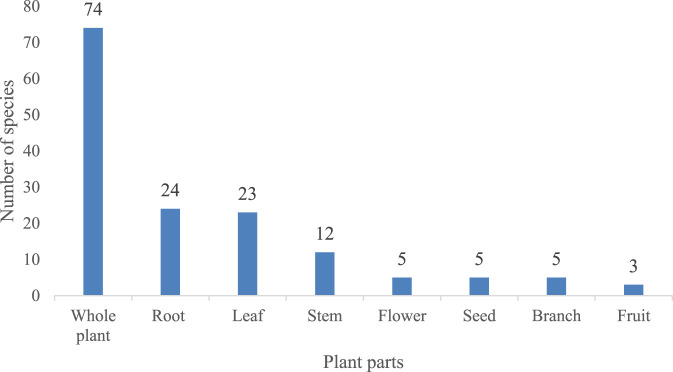
Utilized parts of Lamiaceae plants.

Different parts of the plant are used in different ways and have different therapeutic effects. Respondents reported eight medicinal parts (Figure 2). Among these, the whole plant (74) was the most commonly used form, far exceeding other parts (roots 24, leaves 22, stems 12, etc.). This preference for the whole plant shares similarities with other ethnic medicinal plant utilization patterns. A study in the Jazan region of Saudi Arabia indicated that leaves, fruits, and the whole plant (24%, 18%, and 16%, respectively) are the most commonly used plant parts in the preparation of traditional medicines (Tounekti et al., 2019).

The harvesting season of medicinal plants is critical to their efficacy, as the accumulation of their active compounds is closely linked to environmental factors such as season and climate. Surveys indicate that the harvesting of various parts of Lamiaceae plants is primarily concentrated in the summer and autumn seasons, with only a few harvested in spring and winter. Harvesting locations are diverse, commonly found on slopes (43), grasslands (39), roadside (38), and shrublands (26), among others. This reflects the openness and accessibility of these habitats, as well as the abundance of Lamiaceae plants in these environments. They are also distributed in forests (17), valleys (13), fields (11), and ditches (11).

The Dong ethnic group primarily utilizes Lamiaceae plants for both medicinal and edible purposes. Medicinal use includes internal and external applications. Internally, these plants are commonly prepared as decoctions, teas, or soaked in alcohol, with decoction being the most prevalent method due to its ease of preparation and perceived efficacy. Externally, fresh herbs are frequently pounded and applied directly to affected areas, particularly for treating injuries in field settings. For instance, *Glechoma longituba* is often used in this manner to treat snake bites. Dried herbs are also ground into powder for topical use or infused in water for medicinal bathing.

In addition to their therapeutic roles, many of these plants are incorporated into daily diets, reflecting the concept of medicinal-food homology. Species such as *Agastache rugosa* are commonly consumed as vegetables, with tender stems and leaves used in soups, stir-fries, or cold dishes. These edible plants contribute essential nutrients, including dietary fiber, vitamins, amino acids, and minerals, supporting digestion, immunity, and overall health. Others, like *Mentha spicata*, are valued for their aromatic properties and are used as spices to enhance the flavor and sensory appeal of traditional dishes. For example, *A. rugosa* is added to Dong sour fish soup to deepen its flavor, while chopped mint leaves are used in cold salads to provide a cooling effect and stimulate appetite. These integrated uses highlight the Dong people’s practical knowledge of local plant resources and their ability to align dietary habits with health maintenance.

#### 3.1.3 Specific efficacy of Lamiaceae plants

**FIGURE 3:**
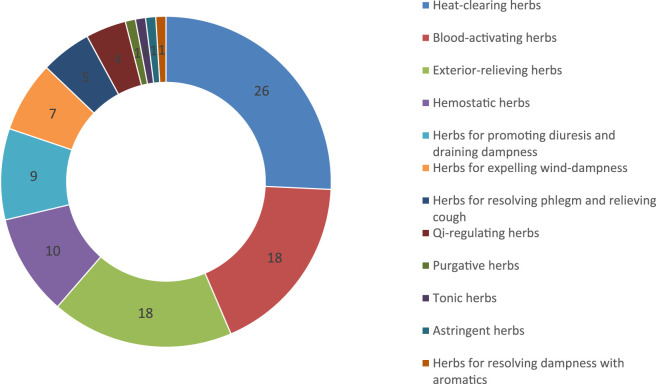
Lamiaceae plants classification according to TMC efficacy in the study area.

According to statistics, the 101 species of Lamiaceae plants investigated can be used to treat respiratory diseases, digestive system diseases, rheumatic diseases, cardiovascular and cerebrovascular diseases, urinary system diseases, inflammations, tonifying the body, endocrine diseases, fractures and so on. These 101 species of Lamiaceae plants were classified and statistically analyzed based on the efficacy of TCM, and were divided into 12 categories in total: 26 species are heat-clearing herbs, 18 species are blood-activating herbs, 18 species are exterior-relieving herbs, 10 species are hemostatic herbs, 9 species are diuretic and dampness-permeating herbs, 7 species are herbs for expelling wind-dampness, 5 species are herbs for resolving phlegm and relieving cough, 4 species are qi-regulating herb, 1 species is purgative herb, 1 species is tonic herb, 1 species is astringent herb, and 1 species is herb for resolving dampness with aromatics (Figure 3). This functional distribution, which focuses on clearing heat and promoting blood circulation, aligns closely with the characteristics of Lamiaceae plants, which are rich in volatile oils, phenolic acids, flavonoids, and other anti-inflammatory and antibacterial compounds. For example, perillaldehyde in perilla leaf volatile oil regulates inflammatory responses by activating the Akt/JNK pathway, while total flavonoids in perilla can address acute inflammation at different stages (Wang et al., 2023).

According to previous studies, different medicinal parts of the same plant also have different efficacies (Qi, 1988). For example, the main medicinal parts of *Perilla frutescens* are its stems, leaves and seeds. *Perilla frutescens* leaves can be used as an antipyretic and antitussive, have the functions of promoting digestion, diuresis, and also possess the effects of relieving pain, calming the nerves and detoxifying. They are used to treat colds and seafood poisoning. The stems of *Perilla frutescens* have the functions of regulating respiration and preventing miscarriage. The seeds of *Perilla frutescens* can relieve cough, resolve phlegm and relieve asthma. This characteristic of “multiple uses from a single plant” and “differential efficacy by plant part” reflects the wisdom of the Dong people in refining plant resource utilization. It also suggests the need to consider the distinct value of different plant parts during development.

#### 3.1.4 The use frequency of Lamiaceae plants by Dong people

**FIGURE 4:**
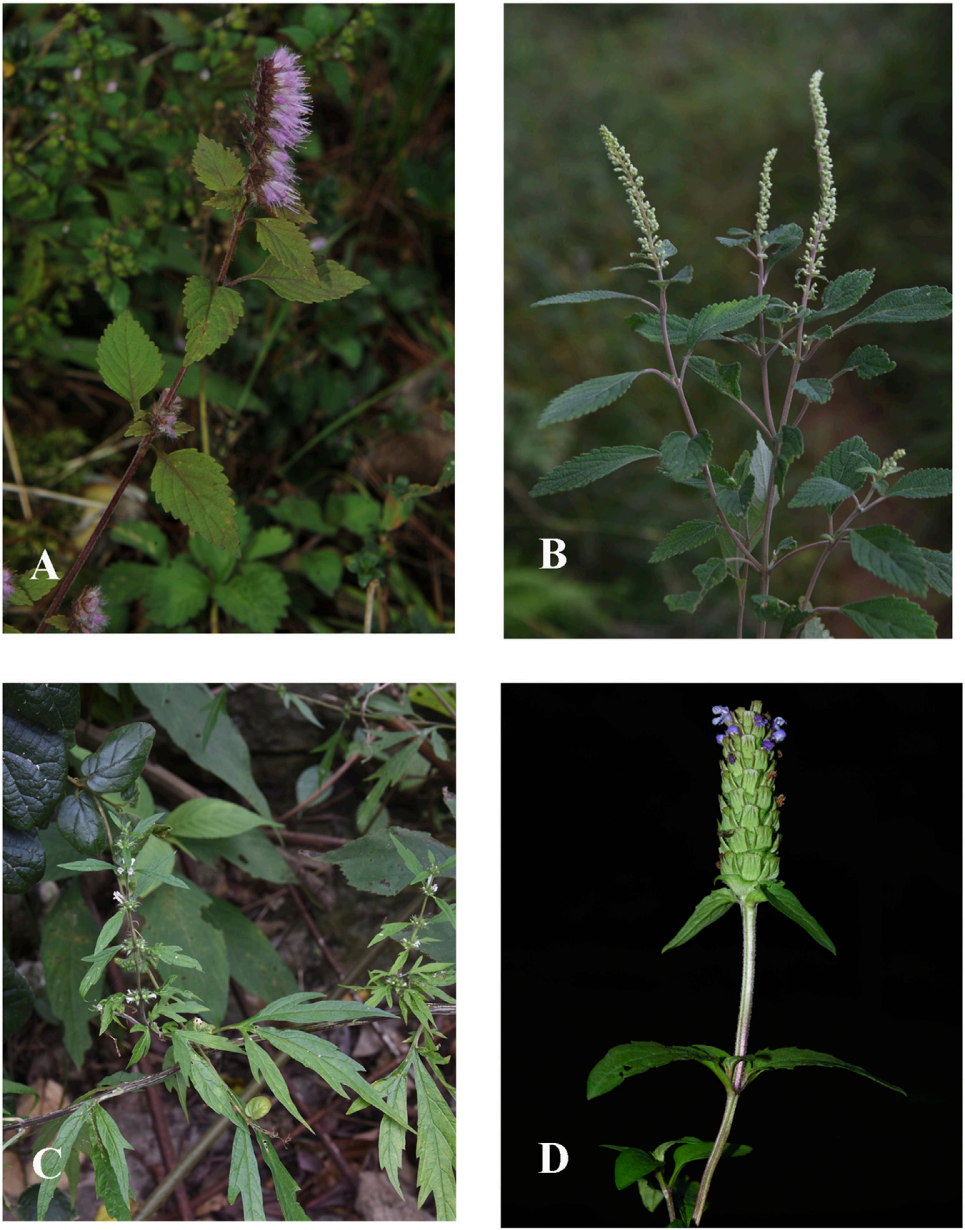
Four frequently used Lamiaceae plants: **(A)** *Elsholtzia ciliata*, **(B)** *Perilla frutescens*, **(C)** *Leonurus japonicus*, **(D)** *Prunella vulgaris*.

The survey found significant differences between Dong men and women in their use of Lamiaceae plants. Notably, contrary to the common observation in many studies that women, as family caregivers, use medicinal plants more frequently (Amar and Lev, 2016), this survey showed that Dong men used Lamiaceae medicinal plants more frequently. We summarized some commonly used plants and their frequency of use (Figure 4).

Men consistently use *G. longituba* and *Clinopodium gracile* to treat traumatic injuries. Additionally, they frequently utilize *A. rugosa*, *Mentha haplocalyx*, *M. spicata*, and *Perilla frutescens*, while *P. vulgaris* is used only occasionally. In contrast, *Lagopsis supina*, *L. japonicus*, and *Leonurus sibiricus*—traditionally used for women’s reproductive health—are rarely or never used by men. This pattern of plant preference is closely tied to gendered labor divisions and health risks. As the primary workforce in agriculture and mountainous forestry, men are more susceptible to physical injuries. *Glechoma longituba* and *C. gracile* are often crushed or chewed and applied directly to wounds, offering an accessible, fast-acting, and effective remedy in field conditions. This ethnobotanical practice represents a pragmatic response to the occupational health challenges associated with physically demanding livelihoods.

In contrast, Dong women’s plant use is more closely associated with reproductive health and domestic responsibilities. *Lagopsis supina*, *L. japonicus*, and *L. sibiricus* are widely used by women to address common gynecological concerns. All female informants reported that they or their female relatives had used these three plants. Specifically, a decoction of *L. supina* is used to relieve menstrual discomfort; *L. japonicus* is taken postpartum to replenish qi and blood; and *L. sibiricus* is employed to alleviate dysmenorrhea. These practices reflect traditional approaches to women’s health that are embedded in everyday life.

In terms of culinary applications, women—drawing on their extensive food knowledge—frequently incorporate *A. rugosa* and *M. spicata* as ingredients or flavoring agents. For instance, *A. rugosa* is added to traditional Dong sour fish soup to enhance its aromatic complexity. *Mentha spicata* is commonly used in desserts, such as crushing its tender leaves and mixing them with glutinous rice flour to make soft, fragrant sweet cakes. This integration of medicinal plants into daily cooking exemplifies the Dong concept of medicinal-food homology, where diet and therapy are harmoniously combined within domestic life.

### 3.2 Results of chemical analysis

The five Lamiaceae species analyzed in this study exhibit distinct physicochemical and compound profiles (Table 1), which reflect their divergent ecological strategies and functional potentials. *Meehania fargesii* showed the highest water content (10.88%), indicating enhanced storage stability and post-harvest freshness. In contrast, *P. vulgaris* had the lowest water content (6.35%) but the highest dry matter accumulation (93.65%), suggesting its suitability for medicinal use due to dense storage of active substances. High dry matter content is typically correlated with greater concentrations of polyphenols, amino acids, and soluble solids, as observed in *Perilla frutescens* (93.57%) and *L. japonicus* (93.2%).

**TABLE 1:** Chemical components of five plants in the Lamiaceae family.

Sample	*Meehania fargesii*	*Prunella vulgaris*	*Leonurus japonicus*	*Perilla frutescens*	*Elsholtzia rugulosa*
Moisture content (%)	10.88 ± 0.15^c^	6.35 ± 0.41^a^	6.8 ± 0.16^a^	6.43 ± 0.24^a^	7.38 ± 0.28^b^
Dry matter content (%)	89.12 ± 0.15^a^	93.65 ± 0.41^c^	93.2 ± 0.16^c^	93.57 ± 0.24^c^	92.62 ± 0.28^b^
Water extract content (%)	27.23 ± 6^bc^	16.58 ± 0.61^a^	29.38 ± 0.35^c^	25.94 ± 0.59^bc^	21.81 ± 3.92^ab^
Free amino acid content (mg/g)	1.54 ± 0.12^c^	0.17 ± 0.01^a^	2.88 ± 0.23^d^	0.28 ± 0.13^ab^	0.61 ± 0.41^b^
Total phenol content (mg/g))	0.8 ± 0.01^a^	0.69 ± 0.02^a^	0.52 ± 0.39^a^	0.83 ± 0.03^a^	0.82 ± 0.04^a^
Total flavonoid content (mg/g)	0.15 ± 0^a^	0.22 ± 0.01^a^	0.21 ± 0.05^a^	0.33 ± 0.11^b^	0.13 ± 0.06^a^
Chlorogenic acid content (mg/g)	0.05 ± 0.02^a^	0.28 ± 0.11^b^	0.03 ± 0^a^	0.04 ± 0.01^a^	0.06 ± 0.02^a^
Rutin content (mg/g)	0.42 ± 0.11^ab^	0.62 ± 0.6^b^	0.04 ± 0.01^a^	0.07 ± 0.09^a^	0.26 ± 0.1^ab^

Note: In the same line, values with the same letter superscripts mean no significant difference (P > 0.05), while with different small letter superscripts mean significant difference (P < 0.05).

*Leonurus japonicus* was particularly rich in water-soluble extracts (29.38%) and free amino acids (2.88 mg/g), indicating strong metabolic activity and promising potential for infusion-based products. In contrast, *E. rugulosa* exhibited moderate extractability (21.81%) and amino acid levels (0.61 mg/g), yet stood out for its high total phenol content (0.82 mg/g), nearly equivalent to *Perilla frutescens* (0.83 mg/g), suggesting considerable antioxidant capacity and health benefits. These phenolic compounds are critical for mitigating oxidative stress and are often key indicators of functional food value.

In terms of flavonoids, *Perilla frutescens* again ranked highest (0.33 mg/g), reinforcing its role in liver protection and anti-inflammatory applications (Ren et al., 2015), while *E. rugulosa* had the lowest content (0.13 mg/g), indicating phenolics as its more dominant bioactive class. *Prunella vulgaris* was notable for its elevated levels of chlorogenic acid (0.28 mg/g) and rutin (0.62 mg/g), both of which far exceeded those in other species, confirming its strong pharmacological potential.

*Elsholtzia rugulosa*, the focal species for tea development, demonstrated a favorable balance of chemical traits: moderate water content (7.38%), high dry matter (92.62%), and strong phenolic presence, alongside a mid-range rutin content (0.26 mg/g)—substantially higher than *L. japonicus* (0.04 mg/g) and *Perilla frutescens* (0.07 mg/g). Although its free amino acid and flavonoid contents are relatively low, its abundant polyphenols and stable dry matter composition offer a solid foundation for development as a functional tea. This compositional profile aligns with its traditional medicinal applications and supports its potential for further processing into health beverages rich in antioxidant compounds.

Together, these findings highlight the chemical diversity among the five Lamiaceae plants and underscore their differentiated potential for development into medicinal or functional tea products. The compound profiles not only explain their ethnobotanical uses but also offer guidance for targeted resource utilization.

### 3.3 Processing and evaluation of *Elsholtzia rugulosa* tea

We produced and evaluated three types of *E. rugulosa* tea (Table 2), and analyzed their key chemical compounds (Table 3). The overall sensory evaluation scores showed no statistically significant differences among the three tea types, suggesting that all methods produced teas of comparable quality.

**TABLE 2:** The evaluation results of three kinds of *Elsholtzia rugulosa* tea.

Different flavors of tea	Appearance and score (20%)	Liquor color and score (10%)	Aroma and score (30%)	Taste and score (40%)	Total score
Taste of green tea	Even; 86	Yellowish-green, Bright; 88	Delicate fragrance, fruity fragrance, Relatively lasting; 89	Mellow; 87	86*20% + 88*10% + 89*30% + 87*40% = 87.5
Taste of oolong tea	Relatively even; 84	Yellowish-orange, Fairly bright; 83	Sweet fruity fragrance, lasting; 87	Relatively mellow with a sweet aftertaste; 88	84*20% + 83*10% + 87*30% + 88*40% = 86.4
Taste of black tea	Even;87	Orange-red, Relatively bright; 85	Characteristic *Elsholtzia rugulosa* fragrance, lasting; 86	Fairly mellow; 84	87*20% + 85*10% + 86*30% + 84*40% = 85.3

**TABLE 3:** Chemical components in *Elsholtzia rugulosa* tea.

Samples/Indicators (%)	Taste of green tea	Taste of oolong tea	Taste of black tea
Total phenols	3.83 ± 0.18	2.76 ± 0.21	4.36 ± 0.03
Total flavonoids	2.97 ± 0.05	1.11 ± 0.01	4.32 ± 0.13
Total free amino acids	2.76 ± 0.03	4.86 ± 0.22	2.28 ± 0.12
Soluble sugars	5.412 ± 0.14	4.615 ± 0.15	6.579 ± 0.12
Water extracts	33.57 ± 0.32	24.08 ± 0.51	32.83 ± 0.33
Moisture	6.22 ± 0.13	5.81 ± 0.17	6.54 ± 0.16
Dry matter	93.78 ± 0.13	94.19 ± 0.17	93.46 ± 0.16

**FIGURE 5:**
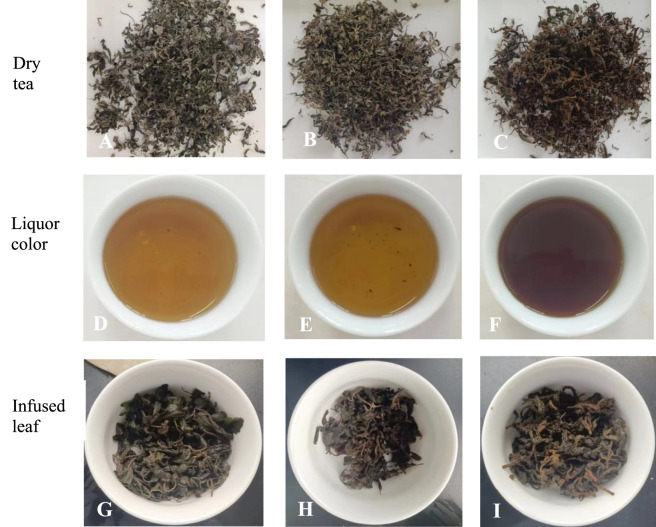
Brewing performance and sensory profiles of *Elsholtzia rugulosa* teas processed by green, oolong, and black tea methods **(A)** Dried green tea leaves, **(B)** Dried oolong tea leaves, **(C)** Dried black tea leaves, **(D)** Green tea infusion, **(E)** Oolong tea infusion, **(F)** Black tea infusion, **(G)** Infused green tea leaves, **(H)** Infused oolong tea leaves, **(I)** Infused black tea leaves.

The processing methods for *E. rugulosa* tea were tailored according to the desired tea type—green, oolong, or black—resulting in distinct outcomes (Figures 5A–I). These variations in processing not only contribute to the unique flavor, aroma, and color profiles of each tea type but also highlight the remarkable versatility of *E. rugulosa* as a tea plant. This diversity enhances both its cultural value and commercial potential, particularly in markets seeking health-oriented and flavor-rich tea products.

In terms of sensory characteristics, *E. rugulosa* green tea produced a yellowish-green infusion, the oolong tea yielded a yellowish-orange infusion, and the black tea presented a rich orange-red color. All three teas exhibited relatively long-lasting aromas. Notably, the black tea infusion retained the characteristic *E. rugulosa* fragrance, while the green and oolong variants developed more fruity notes. Each tea type was described as having a mellow, well-rounded taste, indicating broad potential for consumer acceptance across different flavor preferences.

By analyzing the chemical compounds of the *E. rugulosa* tea soups with the three tastes, it can be known that the *E. rugulosa* black tea ranked first in the contents of total phenols, total flavonoids, soluble sugars and water; the *E. rugulosa* oolong tea ranked first in the contents of total free amino acids and dry matter; and the *E. rugulosa* green tea ranked first in the content of water extracts. It can be concluded from this that the *E. rugulosa* black tea has higher healthcare value and utilization value.

The processing of black tea results in significantly higher total phenolic and total flavonoid contents in *E. rugulosa* tea compared to green tea and oolong tea. This phenomenon is strongly associated with its core processing stage—fermentation. During black tea production, prolonged fermentation is critical. At this stage, disruption of leaf cell structures following rolling facilitates extensive contact between cellular polyphenols (primarily catechins) and atmospheric oxygen. Catalyzed by endogenous polyphenol oxidase and peroxidase, these compounds undergo complex oxidative polymerization reactions.

These newly formed polymeric oxidation products are inherently phenolic compounds and typically exhibit greater chromogenic potential (accounting for the darker infusion color of black tea) and distinct bioactivities compared to their precursor catechins. Consequently, the elevated “total phenolic content” measured in black tea primarily reflects the accumulation of these oxidation polymers, rather than an absolute increase in simple phenolic compounds. This is consistent with the widely studied dynamic change law of polyphenols in the processing of traditional *Camellia sinensis* black tea (Li et al., 2013). Oolong tea, undergoing partial fermentation, exhibits similar but less extensive oxidation, resulting in intermediate phenolic levels between non-fermented green tea and fully fermented black tea. The *E. rugulosa* tea processed using black tea technology in this study exhibited a significantly higher total phenolic content (4.36% ± 0.03%) compared to its green tea (3.83% ± 0.18%) and oolong tea (2.76% ± 0.21%) counterparts. This finding strongly supports the applicability of the principle that the degree of fermentation is a key determinant of polyphenol content and composition in tea products—even in *E. rugulosa*, a non-traditional tea plant. The significant changes in phenolic content and profiles induced by different processing methods, particularly fermentation, provide a scientific basis for explaining the variations in flavor, color, and potential health benefits among tea types.

These insights are especially relevant in the context of urban consumption, where there is growing demand for functional beverages tailored to specific health needs and flavor preferences. By adjusting fermentation levels, *Elsholtzia rugulosa* teas can be diversified to meet the expectations of health-conscious urban consumers seeking natural, plant-based products with distinct sensory experiences and verified bioactivity. This underscores the plant’s potential in expanding the urban tea market with innovative, science-backed herbal tea options.

### 3.4 Limitations of the research scope

The field investigation of this study mainly focused on Yangwei Village (where the Dong population accounted for 95%). Although the sample size of 53 respondents met the methodological requirements of “in-depth interviews with key information providers” in ethnic botany research, it could only reflect the traditional knowledge of this specific community. The geographical environment of Shangzhong Yangwei Village and the cultural made it difficult to directly generalize the research results to the entire Guizhou Province or other ethnic regions.

According to the “Sampling Strategy” principle in “Ethnobiology and Ethnoecology Methods” (Albuquerque et al., 2014), the “random sampling + snowball sampling” combination adopted in this study is suitable for in-depth research of small-scale cultural communities, but its representativeness is limited to the village level. Future research needs to expand the geographical and cultural coverage of the sample through multi-site investigations such as comparisons of different Dong villages in Qiandongnan.

## 4 Conclusion

This study documented the traditional ethnobotanical knowledge of Lamiaceae plants in Yangwei Village (Liping County), Guizhou, mainly among the Dong people, though not exclusively, representing a crucial effort to preserve intangible cultural heritage at risk of being lost. A total of 101 Lamiaceae species from 39 genera were recorded through interviews with 53 local residents and supplemented by extensive field investigation. The Dong’s unique classification system—grouping plants as Ma, Nao, and Nyangt—reflects a culturally embedded understanding of plant function and form. Ethnobotanical data revealed diverse usage methods tailored to specific plant parts and health needs, as well as seasonal harvesting patterns and functional categorizations aligned with traditional Chinese medicine principles. Chemical analysis of five representative species confirmed their medicinal value, with distinct compound profiles supporting their traditional uses and potential as high-value resources for functional food and pharmaceutical development. The study also optimized the processing techniques for *E. rugulosa* tea, resulting in three flavor-adapted tea varieties that meet diverse urban market preferences while retaining health benefits. Future studies expanding to other Dong communities in Guizhou would strengthen regional applicability.

By integrating traditional ethnobotanical knowledge with modern scientific analysis, this research lays the groundwork for translating local plant knowledge into sustainable, health-oriented innovations suitable for urban consumers. It highlights the potential for developing culturally rooted, scientifically supported products that respond to growing urban demand for natural, functional, and personalized wellness options. To realize this potential, coordinated efforts from policymakers, researchers, and industries are essential to support knowledge preservation, responsible resource use, and inclusive community-based development.

## Data Availability

The original contributions presented in the study are included in the article/Supplementary Material, further inquiries can be directed to the corresponding author.
