# Supplementary material for: Ethnobotanical investigations and urban utilization potential of medicinal and edible Lamiaceae plants: a case study from Guizhou, China
Source: Front Pharmacol. 2025 Sep 3;16:1601710. doi: 10.3389/fphar.2025.1601710 (PMC12440854; doi:10.3389/fphar.2025.1601710)
Supplement: Supplementary file 1 [file Table1.docx]

**Supplementary Table 1** Catalogue of 101 medicinal and edible species of Lamiaceae plants

| Local name | Chinese name  (Pinyin) | Growth form | Scientific name | Part used | Preparation method | Collection location | Traditional uses |
| --- | --- | --- | --- | --- | --- | --- | --- |
| Yu xiang | Huo xiang | Herb | *Agastache rugosa* (Fisch. et C. A. Mey.) Kuntze | Whole plant, Fruit, Leaves, Stems | Decoction, Making spices, Producing aromatic oils | Field, Roadside | Relieve vomiting, treat abdominal pain in cholera, and clear away summer-heat, the fruit can be used as a spice |
| Ku cao | Jin guo cao | Herb | *Ajuga ciliata* Bunge | Whole plant | Decoction, Apply after pounding | Roadside, Riverbank, At the foot of the mountain, Wasteland | Treat hemoptysis caused by lung heat, traumatic injuries, tonsillitis and pharyngitis |
| Bai mao xia ku cao | Jin chuang xiao cao | Herb | *Ajuga decumbens* Thunb. | Whole plant | Decoction, Apply after pounding, Decoct for washing | Field, Grassland, Roadside | Treat carbuncles, furuncles, pharyngitis, gastroenteritis, acute conjunctivitis, burns, dog bites, venomous snake bites and other symptoms |
| San xue cao | Da zi jin gu | Herb | *Ajuga macrosperma* Wall. ex Benth. | Whole plant | Decoction | Roadside, Grassland | It is used for dispersing blood stasis |
| Shi hui cai | Zi bei jin pan | Herb | *Ajuga nipponensis* Makino | Whole plant | Decoction, Apply after pounding | Field, Forests, On the sunny slopes | Treat pneumonia, tonsillitis, pharyngitis, tracheitis, mumps, and acute cholecystitis. |
| Fang feng cao | Guang fang feng | Herb | *Anisomeles indica* (L.) Kuntze | Whole plant | Decoction, Infuse in wine, Apply after pounding, Decoct for washing | At the edge of the forest, Roadside | Treat rheumatic bone pain, fever caused by cold, vomiting, abdominal pain, skin eczema, and bites from venomous insects |
| Yang er duo | Da ye zi zhu | Shrub | *Callicarpa macrophylla* Vah | Leaves, Roots | Decoction, Apply after pounding | Streamside, Roadside | Treat traumatic swelling and pain, wound bleeding, intestinal bleeding, hemoptysis and epistaxis |
| Local name | Chinese name  (Pinyin) | Growth form | Scientific name | Part used | Preparation method | Collection location | Traditional uses |
| Xiao hong mi guo | Hong zi zhu | Shrub | *Callicarpa rubella* Lindl. | Leaves, Roots, Buds | Decoction, Apply by pounding | Mountain slopes, River valleys, Forests, Bushes | Promote menstrual flow and treat abnormal vaginal discharge in women |
| Ai tong zi | Chou mu dan | Shrub | *Clerodendrum bungei* Steud. | Roots, Stems, Leaves | Decoction, Apply by pounding, Wash by decocting in water | Near the moist forest edges, Mountain valleys, Beside houses. | Dispel wind and detoxify, reduce swelling and relieve pain, and treat uterine prolapse |
| Da qing | Da qin | Shrub | *Clerodendrum cyrtophyllum* Turcz. | Leaves, Roots | Decoction, Apply after pounding, Decoct for washing | Seaside, Forest, Bushes beside the water, Plain, Hills, Mountain forest, Valleys | Clear heat, purge fire, promote diuresis, cool the blood, and resolve toxins |
| Chou tong | Hai zhou chang shan | Shrub | *Clerodendrum trichotomum* Thunb. | Roots, Stems, Leaves | Decoction, Apply after pounding | Mountain slopes, Bushes | Treat rheumatic arthralgia, hemiplegia, migraine, and malaria |
| Jian dao cao | Xi feng lun cai | Herb | *Clinopodium gracile* (Benth.) Matsum. | Whole plant | Decoction, Apply after pounding, Decoct for washing | Roadside, Ditches, Grassland, Forest | Treat headache due to cold, dysentery, mastitis, carbuncles and swollen toxins, urticaria, allergic dermatitis |
| She chuang zi | Cun jin cao | Herb | *Clinopodium megalanthum* (Diels) C. Y. Wu & S. J. Hsuan ex H. W. Li | Whole plant | Decoction | Mountains slopes, Valleys, Thickets, Gravelly grasslands | Treat toothache, infantile malnutrition, rheumatism - related injuries, reduce swelling and promote blood circulation. |
| Local name | Chinese name  (Pinyin) | Growth form | Scientific name | Part used | Preparation method | Collection location | Traditional uses |
| Shan huo xiang | Deng long cao | Herb | *Clinopodium polycephalum* (Vaniot) C. Y. Wu & S. J. Hsuan ex P. S. Hsu | Whole plant | Decoction, Apply after pounding, Decoct for washing | Mountain slopes, Roadsides, Forests, Thickets. | Treat functional uterine bleeding, cholecystitis, jaundice - type hepatitis, headache caused by cold, hemorrhoid |
| Hong dian | Mao e qiao rui hua | Herb | *Coleus esquirolii* (H. Lév.) Dunn | Whole plant | Decoction, Apply after pounding, Grind into powder | Rocky mountains, Beside rocks in valleys, Grassy slopes | Stop bleeding and promote bone union |
| Ye yu xiang | Mian sui su | Herb | *Comanthosphace ningpoensis* (Hemsl.) Hand.-Mazz. | Whole plant | Decoction, Apply after pounding | Mountains slopes, Grassland, Beside the brooks | Treat paralysis, colds, headaches, and hematemesis |
| Xiang gu | Xiang ru | Herb | *Elsholtzia ciliata* (Thunb.) Hyl. | Whole plant | Decoction, Make into pills, Apply by pounding | Roadside, Mountains slopes, Wasteland, Forest, Riverbanks | Treat acute gastroenteritis, abdominal pain with vomiting and diarrhea, headache accompanied by fever |
| Xiang zi su | Ji long cao | Herb | *Elsholtzia communis* (Coll. et Hemsl.) Diels | Stems, Leaves | Decoction | Riverside, Roadside, Valleys, Forest, Thickets, Ditches | Treat colds, headaches, fever and indigestion |
| Local name | Chinese name  (Pinyin) | Growth form | Scientific name | Part used | Preparation method | Collection location | Traditional uses |
| Ye ba zi | Ye ba zi | Herb | *Elsholtzia rugulosa* Hemsl. | Branches, Leaves | Decoction, Make tea | Grasslands of mountain slopes, Roadside, Forest, Thickets | Used to treat colds, indigestion, abdominal pain and distension, and gastroenteritis |
| Mu jiang zi | Chuan dian xiang ru | Herb | *Elsholtzia souliei* H. Lév. | Whole plant | Decoction | Mountains slopes, Grassland | Used to treat infantile convulsions |
| Su ma | Qiu sui xiang ru | Herb | *Elsholtzia strobilifera* Benth | Whole plant | Decoct to make a thick paste | Mountains slopes, Grasslands, Valleys, Forest, Thickets | It is used as a medicinal for promoting eruption |
| Jie guo xiao | Huo xue dan | Herb | *Glechoma longituba* (Nakai) Kuprian. | Whole plant, Stems, Leaves | Decoction, Apply after pounding | Forest, Grassland, Beside the brook. | Treat bladder stones or urinary tract stones. For external use, it can be applied to traumatic injuries and fractures. |
| Ye zhi ma | Xi chi yi ye zhi ma | Herb | *Heterolamium debile* var. *cardiophyllum* (Hemsl.) C. Y. Wu | Whole plant | Decoction, Apply after pounding | Under the forest, Bamboo groves, Ditches, Grassy slopes, Forest edges. | Treat smallpox. |
| Niu xi cao | Shen xiang cao | Semi-shrub | *Hyssopus officinalis* L. | Whole plant | Decoction | Stony mountains, Arid grasslands | The oil is mainly used as a spice for sweet wine. |
| Shui long dan | Xian hua xiang cha cai | Herb | *Isodon adenanthus* (Diels) Kudô | Roots | Decoction, Grind into power | Under pine forests, Pine-oak forests, Bamboo forests | Treat dyspepsia, gastroenteritis and dysentery. |
| Local name | Chinese name  (Pinyin) | Growth form | Scientific name | Part used | Preparation method | Collection location | Traditional uses |
| Sui lan hua | Xian wen xiang cha cai | Herb | *Isodon lophanthoides* (Buch.-Ham. ex D. Don) H. Hara | Whole plant | Decoction, Apply after pounding, Grind into powder | Marsh, Damp areas under the forest | Treat acute icteric hepatitis and acute cholecystitis. |
| Dou gan sha | Da zhui xiang cha cai | Herb | *Isodon megathyrsus* (Diels) H. W. Li | Roots | Decoction | Pine-oak forests and spruce forests in valleys | Stop bleeding and regulate qi. |
| Lan hua chai hu | Xian mai xiang cha cai | Herb | *Isodon nervosus* (Hemsl.) Kudô | Stems, Leaves | Decoction, Apply by pounding, Wash by decocting in water | Forest, Grassland | Treat acute infectious hepatitis, venomous snake bites, and impetigo. |
| Ye huo xiang | Yin hua xiang cha cai | Herb | *Isodon rosthornii* (Diels) Kudô | Whole plant | Decoction, Apply by pounding, Infuse in wine | Mountains slopes | Dispel cold, promote sweating, clear heat, resolve phlegm and reduce swelling. |
| Dong lin cao | Sui mi ya | Shrub | *Isodon rubescens* (Hemsl.) H. Hara | Whole plant | Decoction, Grind into powder, Infuse in wine, Soak in oil for topical application | Mountains slopes, Bushes, Woodlands, Gravelly areas and sunny places beside roads | Treat cold - induced headache, rheumatic pains in muscles and bones, and joint pain. |
| Xi gou cao | Xi huang cao | Herb | *Isodon serra* (Maxim.) Kudô | Whole plant | Decoction, Apply by pounding | Mountains slopes, Roadsides, Fields, Beside brooks, Riverbanks, Grasslands, Thickets | Treat acute hepatitis, acute cholecystitis, and bruises and swelling from falls and contusions. |
| Local name | Chinese name  (Pinyin) | Growth form | Scientific name | Part used | Preparation method | Collection location | Traditional uses |
| Chong ya cao | Niu wei cao | Herb | *Isodon ternifolius* (D. Don) Kudô | Whole plant, Leaves | Decoction, Apply by pounding, Grind into powder | Grasslands, Thickets | Treat dysentery, enteritis and jaundice hepatitis, grind the leaves for external application to treat impetigo. |
| Deng long ke | Xia zhi cao | Herb | *Lagopsis supina* (Steph.) Ikonn.-Gal. | Whole plant | Decoction, Decoct to make a thick paste | Roadside | For internal use, it can treat dysmenorrhea and menstrual disorders in women. Tender seedlings used as medicine have the effect of enriching the blood. The flowers can treat anemia and weakness. |
| Jie gu cao | Bao gai cao | Herb | *Lamium amplexicaule* L. | Whole plant | Apply by pounding | Roadsides, wastelands. | Treat traumatic fractures, injuries from falls, bumps and contusions with swelling and redness. |
| Shan su zi | Ye zhi ma | Herb | *Lamium barbatum* Siebold & Zucc. | Whole plant, Flower | Decoction, Apply by pounding | Roadside, Brook, On the ridges of fields, Barren slopes | The flowers are used to treat uterine and urinary system disorders, the Whole plant is used for treating traumatic injuries and infantile malnutrition. |
|  | Bai hua yi mu cao | Herb | *Leonurus artemisia* var.*albiflorus* (Migo) S.Y.Hu. | Whole plant, Seeds, Flower, Seedings | Decoction, Decoct to make a thick paste, Make into pills, Apply by pounding | Mountains slopes, Hill | For internal use, it can treat dysmenorrhea and menstrual disorders in women. Tender seedlings used as medicine have the effect of enriching the blood. The flowers can treat anemia and weakness. |
| Deng long cao | Yi mu cao | Herb | *Leonurus japonicus* Houtt. | Whole plant, Seeds, Flower, Seedings | Decoction, Decoct to make a thick paste, Make into pills, Apply by pounding | Grassland, Streamside, Roadside | For internal use, it can treat dysmenorrhea and menstrual disorders in women. Tender seedlings used as medicine have the effect of enriching the blood. The flowers can treat anemia and weakness. |
|  | Zan cai | Herb | *Leonurus pseudomacranthus* Kitag. | Whole plant | Decoction, Apply by pounding, Grind into power | On the ridges of fields, Roadside, Beside brooks | Treat postpartum abdominal pain. |
| Local name | Chinese name  (Pinyin) | Growth form | Scientific name | Part used | Preparation method | Collection location | Traditional uses |
|  | Xi yi mu cao | Herb | *Leonurus sibiricus* L. | Whole plant, Fruits | Decoction, Apply after pounding | By the stream, Mountains | Nourish the body |
| Pao hua cao | Xiu qiu fang feng | Herb | *Leucas ciliata* Hochst. ex Benth. | Whole plant, Fruit, Roots | Decoction, Apply by pounding, Grind into power | Beside brooks, Valleys, Roadside, Thickets, Grassland | The roots are used to treat rheumatic numbness, pain and rashes. The fruits are used to treat colds caused by wind - cold and infantile pneumonia. The Whole plant is used to treat sores, ulcers, swelling and toxicity. |
| [Bei feng cao](https://www.iplant.cn/info/%E5%8C%97%E9%A3%8E%E8%8D%89) | Bai rong cao | Herb | *Leucas mollissima* Wall. | Whole plant | Apply by pounding | Thickets, Roadsides, Grasslands, Beside brooks | Treat kidney deficiency. |
|  | Bai rong cao shu mao bian zhong | Herb | *Leucas mollissima* var. *chinensis* Benth. | Whole plant | Grind into powder, Apply by pounding | Flatlands, hills | Expel cold and relieve the exterior syndrome. For external use, it can treat sore toxins. |
| Di gua miao er | Ying mao di sun | Herb | *Lycopus lucidus* var. *hirtus* Regel | Whole plant, Roots | Decoction, Stir-fried roots | Swamps, watersides | The Whole plant can promote menstruation and diuresis, the root is edible and can be used to treat metal - induced sores, swelling, toxicity, rheumatic joint pain. |
| Shui shen ma | Hua xi long tou cao | Herb | *Meehania fargesii* (H. Lév.) C. Y. Wu | Whole plant | Decoction | Forest | Relieve the exterior syndrome and dispel cold, diffuse the lung qi and relieve cough. |
|  | Hua xi long tou cao geng hua bian zhong | Herb | *Meehania fargesii* var. *pedunculata* (Hemsl.) C. Y. Wu | Whole plant | Decoction | Forest | Treat diarrhea. |
| Local name | Chinese name  (Pinyin) | Growth form | Scientific name | Part used | Preparation method | Collection location | Traditional uses |
|  | Hua xi long tou cao zou jing bian zhong | Herb | *Meehania fargesii* var. *radicans* (Vaniot) C. Y. Wu | Whole plant | Decoction | Forest | Treat colds caused by wind - cold, and also applied externally to treat snake bites. |
| Li yu cao | Long tou cao | Herb | *Meehania henryi* (Hemsl.) Sun ex C. Y. Wu | Roots | Decoction, Apply by pounding | Forest | For snake bites, apply the leaves externally. For tonifying blood, soak the roots in wine and take it. |
| Tu jin jie | Mi feng hua | Herb | *Melissa axillaris* (Benth.) Bakh. F. | Whole plant | Decoction, Wash by decocting in water, Apply by pounding | Roadsides, Mountains, Mountains slopes, Valleys | Treat epistaxis, dysentery and snake bites. |
| Tu bo he | Bo he | Herb | *Mentha canadensis* L. | Tender stem tips, Whole plant | Decoction, Make into pills, Apply by pounding | Mountains and plains, Roadside, Village, Wilderness, Behind the house | Treat cold, fever, sore throat and headache. The tender stem tips can be used as vegetables for food. |
|  | Zhou ye liu lan xiang | Herb | *Mentha crispata* Schrad. ex Willd. | Tender branches and leaves | Decoction, Make spices | Roadside | Tender branches and leaves are used as spices for consumption. |
| [Xiang hua cai](https://www.iplant.cn/info/%E9%A6%99%E8%8A%B1%E8%8F%9C) | Liu lan xiang | Herb | *Mentha spicata* L. | Whole plant, Tender branches, Leaves | Decoction, Apply by pounding | Roadside | Treat cold, fever, cough, and consumptive cough. Tender branches and leaves are used as flavoring spices for consumption. |
| Local name | Chinese name  (Pinyin) | Growth form | Scientific name | Part used | Preparation method | Collection location | Traditional uses |
| [Xiao jiang cao](https://www.iplant.cn/info/%E5%B0%8F%E5%A7%9C%E8%8D%89) | Jiang wei cao | Subshrub | *Micromeria biflora* (Buch.-Ham. ex D. Don) Benth. | Whole plant | Decoction, Grind into powder | Limestone slopes, sunny grassy slopes. | Treat stomachache, abdominal distension, vomiting and diarrhea. |
| Ye xiang ru | Xiao hua ji zhu | Herb | *Mosla cavaleriei* H. Lév. | Whole plant | Decoction, Apply by pounding | Sparse forests, Mountains slopes, Grasslands | Treat heatstroke with fever and cold with aversion to cold, for external use, treat venomous snake bites. |
| Xiang ru cao | Shi xiang ru | Herb | *Mosla chinensis* Maxim. | Whole plant | Decoction, Grind into powder, Decoct for washing, Apply by pounding | Wastelands, Roadsides, Edges of fields, Grasslands on slopes | Treat heatstroke with fever and cold with aversion to cold, for external use, treat venomous snake bites. |
| Yue wei cao | Xiao yu xian cao | Herb | *Mosla dianthera* (Buch.-Ham. ex Roxb.) Maxim. | Whole plant | Decoction, Apply after pounding, Decoct for washing | Under the bushes on slopes and beside ditches | Treat inflammatory edema, traumatic bleeding and other symptoms. It can kill mosquitoes. |
|  | Shi jie zhu | Herb | *Mosla scabra* (Thunb.) C. Y. Wu et H. W. Li | Whole plant, Roots | Decoction, Apply by pounding | Mountains slopes, Roadsides, Thickets | Treat colds, heatstroke with high fever, itchy skin and kill insects, the roots can treat sores and toxins. |
| [Zhang nao cao](https://www.iplant.cn/info/%E6%A8%9F%E8%84%91%E8%8D%89) | Jin jie | Herb | *Nepeta cataria* L. | Whole plant | Decoction, Apply by pounding, Decoct for washing, Grind into power | Mountains slopes, Beside ditches and ponds, Thickets | Prevent and treat colds. |
| Local name | Chinese name  (Pinyin) | Growth form | Scientific name | Part used | Preparation method | Collection location | Traditional uses |
|  | Luo le shu rou mao bian zhong | Herb | *Ocimum basilicum* L. | Whole plant, Tender leaves, Stems, Leaves, Seeds | Decoction, Squeeze out juice, Make into pills, Make tea, Make spices | Roadside, Ditches, Grassland, Forest | Aromatic ingredients；The tender leaves are edible, which has a diaphoretic effect. The Whole plant is used to treat stomachache and gastric spasm, the stems and leaves can promote blood circulation, and the seeds can be used for contraception. |
| [Sheng tou cao](https://www.iplant.cn/info/%E7%9C%81%E5%A4%B4%E8%8D%89) | Luo le | Herb | *Ocimum basilicum* var. *pilosum* (Willd.) Benth. | Whole plant, Tender leaves, Stems, Leaves, Seeds | Decoction, Squeeze out juice, Make into pills, Make tea, Make spices | Roadside, Ditches, Grassland, Forest | Aromatic ingredients；The tender leaves are edible, which has a diaphoretic effect. The Whole plant is used to treat stomachache and gastric spasm, the stems and leaves can promote blood circulation, and the seeds can be used for contraception. |
| [Wu xiang cao](https://www.iplant.cn/info/%E4%BA%94%E9%A6%99%E8%8D%89) | Niu zhi | Herb | *Origanum vulgare* L. | Whole plant | Decoction, Make tea, Apply by pounding | Mountains slopes, Grasslands, Forest, | Prevent influenza and treat heatstroke as well as colds. |
| [Hua chong xiao](https://www.iplant.cn/info/%E5%8C%96%E8%99%AB%E6%B6%88) | Ji jiao shen | Herb | *Orthosiphon wulfenioides* (Diels) Hand.-Mazz. | Roots | Decoction, Grind into powder | Mountains slopes, Grassland | Treat indigestion, food stagnation, ascariasis, and rheumatic pain. |
|  | Ji jiao shen jing ye bian zhong | Herb | *Orthosiphon wulfenioides* var. *foliosus* E. Peter | Roots | Decoction, Grind into powder | Mountains slopes, Grassland | Treat indigestion, food stagnation, ascariasis, and rheumatic pain. |
|  | Zi su | Herb | *Perilla frutescens* (L.) Britton | Stems, Leaves, Seeds | Decoction, Grind into power, Apply by pounding | Field, Ditchside | The leaves can sedation and detoxification, the stems can regulate qi and prevent miscarriage, the seeds can relieve and asthma. Seeds can be pressed to extract oil. |
| Local name | Chinese name  (Pinyin) | Growth form | Scientific name | Part used | Preparation method | Collection location | Traditional uses |
|  | Cao su nan fang bian zhong | Herb | *Phlomis umbrosa* var. *australis* Hemsl. | Whole plant, Roots | Decoction | The edge of forests, Grasslands, Roadsides | Treat gastroenteritis, pneumonia, colds, and coughs caused by tuberculosis. |
| [Shan zhi ma](https://www.iplant.cn/info/%E5%B1%B1%E8%8A%9D%E9%BA%BB) | Cao su | Herb | *Phlomoides umbrosa* (Turcz.) Kamelin & Makhm. | Roots | Decoction | The edge of forests, Grasslands, Roadsides | It can reduce swelling, promote tissue regeneration, repair damaged tendons and prevent miscarriage. |
| [Ji gu tou cai](https://www.iplant.cn/info/%E9%B8%A1%E9%AA%A8%E5%A4%B4%E8%8F%9C) | Mo ye ci rui cao | Herb | Nepeta cataria L. | Branches, Leaves | Decoction | Valleys, Beside brooks, Roadsides | Treat uterine prolapse. |
| [Dou fu tou](https://www.iplant.cn/info/%E8%B1%86%E8%85%90%E8%8D%89) | Dou fu chai | Shrub | *Premna microphylla* Turcz | Roots, Leaves, Stems | Decoction, Apply by pounding, Decoct for washing, Grind into power | Mountain slopes, Forests, Roadside | Treat malaria, diarrhea and dysentery. |
| [Deng long tou](https://www.iplant.cn/info/%E7%81%AF%E7%AC%BC%E5%A4%B4) | Shan bo cai | Herb | *Prunella asiatica* Nakai | Whole plant | Decoction, Make tea | Roadsides, Grasslands on slopes, Thickets, Damp ground | It has diuretic and blood pressure-lowering effects, can treat gonorrhea and scrofula, and can also be used as a tea beverage. |
|  | Xia ku cao | Herb | *Prunella vulgaris* L. | Whole plant | Decoction, Decoct to make a thick paste, Make into pills, Apply by pounding | Understory, Brush, Roadside | Treat facial paralysis (crooked mouth and eyes) and relieve pain in the bones and muscles. |
| Local name | Chinese name  (Pinyin) | Growth form | Scientific name | Part used | Preparation method | Collection location | Traditional uses |
|  | Mi die xiang | Subshrub | *Rosmarinus officinalis* L. | Roots, Leaves, Stems | Make spices | Roadside, Slopes of mountains, Wasteland, Forest, Riverbanks | It can be used as a blending raw material for soap or cosmetic fragrances. |
|  | Cheng se shu wei cao | Herb | *Salvia aerea* H. Lév. | Roots | Decoction | Grasslands, Forests, Bushes, Mountain slopes | Treat dizziness and rheumatic pain, tonify the kidneys. |
| [Hong qing cai](https://www.iplant.cn/info/%E7%BA%A2%E9%9D%92%E8%8F%9C) | Xue pen cao | Herb | *Salvia cavaleriei* var. *simplicifolia* E. Peter | Whole plant, Leaves | Decoction, Apply after pounding | Mountains slopes, Forest, Ditches | Treat spitting blood and metrorrhagia. The leaves can be applied externally to treat sores and toxins. |
| [Huo xue cao](https://www.iplant.cn/info/%E6%B4%BB%E8%A1%80%E8%8D%89) | Hua shu wei cao | Herb | *Salvia chinensis* Benth. | Whole plant, Roots | Decoction, Apply by pounding, Extract juice by wringing | Mountains slopes, Grassland, Roadside | Treat hepatitis and dysmenorrhea. When the roots are soaked in liquor and taken orally, it can regulate menstruation and promote blood circulation. |
|  | Mao di shu wei cao | Herb | *Salvia digitaloides* Diels | Roots | Decoction | Under the mountain pine forests, Mountains slopes, Grasslands | Promote blood circulation to remove blood stasis, and relieve pain by dredging the channels. |
| [Xue shen](https://www.iplant.cn/info/%E8%A1%80%E5%8F%82) | Dan shen | Herb | *Salvia miltiorrhiza* Bunge | Roots, Stems | Infuse into wine | Sunny places in the mountains and fields | Treat liver cirrhosis. |
| Local name | Chinese name  (Pinyin) | Growth form | Scientific name | Part used | Preparation method | Collection location | Traditional uses |
| [Ha ma pi](https://www.iplant.cn/info/%E8%9B%A4%E8%9F%86%E7%9A%AE) | Li zhi cao | Herb | *Salvia plebeia* R. Br. | Whole plant | Decoction, Apply by pounding, Extract juice by wringing | Mountain slopes, Roadsides, Wastelands, Wetlands beside rivers. | For treating traumatic injuries, swelling and pain of the throat, and infantile convulsions. |
| [Shan hu jiao](https://www.iplant.cn/info/%E5%B1%B1%E8%83%A1%E6%A4%92) | Chang guan shu wei cao | Herb | *Salvia plectranthoides* Griff. | Whole plant, Roots | Decoction | Mountain slopes, Sparse forest | Treat common cold and abdominal pain. The roots soaked in liquor can be used to treat coughs caused by colds. |
| [Hong di dan](https://www.iplant.cn/info/%E7%BA%A2%E5%9C%B0%E8%83%86) | Hong geng cao | Herb | *Salvia prionitis* Hance | Whole plant | Decoction, Grind into powder | Mountain slopes, Sunny grasslands | Treat bacillary dysentery, diarrhea, abdominal pain and colds. |
| [Tian qin cai](https://www.iplant.cn/info/%E7%94%B0%E8%8A%B9%E8%8F%9C) | Di geng shu wei cao | Herb | *Salvia scapiformis* Hance | Whole plant | Decoction, Infuse into wine | Behind the house, Mountains and plains | Treat lung diseases. |
| [Hu guang cao](https://www.iplant.cn/info/%E6%B9%96%E5%B9%BF%E8%8D%89) | Fo guang cao | Herb | *Salvia substolonifera* E. Peter | Whole plant | Decoction, Apply after pounding, Stewed meat | Forest, Ditches, Crevices of rocks | Treat coughs caused by overexertion, wheezing coughs, and spitting blood. |
| [Xiao hong shen](https://www.iplant.cn/info/%E5%B0%8F%E7%BA%A2%E5%8F%82) | Yun nan shu wei cao | Herb | *Salvia yunnanensis* C. H. Wright | Roots | Decoction, Apply after pounding, Make into pills | Mountain slopes, Roadsides, Forests | Promote blood circulation to remove blood stasis, and relieve pain by dredging the channels. |
| [Xiao hui xiang](https://www.iplant.cn/info/%E5%B0%8F%E8%8C%B4%E9%A6%99) | Lie ye jin jie | Herb | *Schizonepeta tenuifolia* (Benth.) Briq. | Whole plant, Flower spike | Decoction, Apply after pounding, Make into pills, Decoct for washing | The edge of pine forests, Mountains slopes, Grasslands | Treat colds caused by wind - cold, headache, and sore throat. |
| Local name | Chinese name  (Pinyin) | Growth form | Scientific name | Part used | Preparation method | Collection location | Traditional uses |
|  | Dian huang cen | Herb | *Scutellaria amoena* C. H. Wright | Roots, Stems | Decoction, Make tea | Under the pine forest, Grassland | Treat coughs due to lung heat and swelling and pain of red eyes. The stems and leaves can be used as a substitute for tea for drinking. |
| [Bin tou cao](https://www.iplant.cn/info/%E5%B9%B6%E5%A4%B4%E8%8D%89) | Ban zhi lian | Herb | *Scutellaria barbata* D. Don | Whole plant | Decoction, Decoct for washing | Beside brooks, Beside farmland, Moist grasslands | Treat various inflammations, traumatic injuries, and insect bites. |
| [Yi zhi jian](https://www.iplant.cn/info/%E4%B8%80%E6%94%AF%E7%AE%AD) | Yi se huang cen | Herb | *Scutellaria discolor* Wall. ex Benth. | Whole plant | Decoction | Under the mountain forests, Beside brooks, Grassy slopes | Treat colds, high fever, and gastroenteritis. |
| [Da li cao](https://www.iplant.cn/info/%E5%A4%A7%E5%8A%9B%E8%8D%89) | Han xin cao | Herb | *Scutellaria indica* L. | Whole plant | Decoction, Apply by pounding, Infuse in wine | Mountainous, Hilly, Under sparse forests, Roadsides Grasslands | Treat traumatic injuries, expel wind, and strengthen the muscles and bones. |
|  | Dun ye huang cen | Herb | *Scutellaria obtusifolia* Hemsl. | Whole plant | Decoction | Forests, Bushes, Wet areas beside paddy fields | Treat diarrhea, bitter taste in the mouth, and colds. |
| [Si xiang hua](https://www.iplant.cn/info/%E5%9B%9B%E9%A6%99%E8%8A%B1) | Si lie hua huang cen | Herb | *Scutellaria quadrilobulata* Y. Z. Sun ex C. H. Hu | Whole plant | Decoction | Mountains slopes, Bushes | It has the functions of clearing the liver and relieving the exterior syndrome. |
| [Hu dou cao](https://www.iplant.cn/info/%E8%83%A1%E8%B1%86%E8%8D%89) | Shi wo gong cao | Herb | *Scutellaria sessilifolia* Hemsl. | Whole plant | Decoction, Apply after pounding | Forest, Bushes, Damp rocky mountains | Treat dizziness caused by colds, tinnitus due to liver heat, and eliminate swelling and toxins. |
| Local name | Chinese name  (Pinyin) | Growth form | Scientific name | Part used | Preparation method | Collection location | Traditional uses |
| Tu huang cen | Pian hua huang cen | Herb | *Scutellaria tayloriana* Dunn | Roots | Decoction | Forest, Bushes | Treat coughs caused by heat, spitting blood and dysentery with bloody stools. |
|  | Hong jing huang cen | Herb | *Scutellaria yunnanensis* H. Lév. | Whole plant | Decoction, Apply by pounding | Forest, Beside the valleys and ditches | Treat eye heat, nebula, and can reduce fever. |
|  | Liu ye hong jing huang cen | Herb | *Scutellaria yunnanensis* var. *salicifolia* Y. Z. Sun ex C. H. Hu | Whole plant | Decoction | Beside the valleys, Bushes | It has the effect of reducing internal heat. |
| [Cao teng wu](https://www.iplant.cn/info/%E8%8D%89%E8%97%A4%E4%B9%8C) | Tong guan hua | Herb | *Siphocranion macranthum* (Hook. f.) C. Y. Wu | Stems, Leaves | Decoction, Apply after pounding | Growing in evergreen forests or mixed forests | Treat sores and toxins. |
| Zhi ma cao | Shui su | Herb | *Stachys japonica* Miq. | Whole plant, Roots | Decoction, Decoct for washing, Grind into power, Apply by pounding | Beside the fields and ditches | Treat tonsillitis, pharyngolaryngitis, and the roots can cure herpes zoster. |
| [Shui hui xiang](https://www.iplant.cn/info/%E6%B0%B4%E8%8C%B4%E9%A6%99) | Zhen tong cai | Herb | *Stachys oblongifolia* Benth. | Whole plant | Decoction | Forest, Along the riverbank, Bamboo thickets, Bushes, Red beds, Grasslands, Wetlands | Treat chronic dysentery, weakness caused by long-term illness and bleeding from external injuries. |
| Local name | Chinese name  (Pinyin) | Growth form | Scientific name | Part used | Preparation method | Collection location | Traditional uses |
| [Luo si cai](https://www.iplant.cn/info/%E8%9E%BA%E8%9B%B3%E8%8F%9C) | Gan lu zi | Herb | *Stachys sieboldii* Miq. | Stems, Whole plant | Decoction, Stir-fried | Wetlands, Waterlogged areas | The tubers are used to make pickles. The whole herb is used to treat pneumonia and wind-heat colds. |
|  | An long xiang ke ke | Herb | *Teucrium anlungense* C. Y. Wu et S. Chow | Whole plant | Decoction | Under the mountain forest | Treat infantile indigestion and abdominal distension. |
|  | Er chi xiang ke ke | Herb | *Teucrium bidentatum* Hemsl. | Roots | Decoction | Under the mountain forest | Treat dysentery and leukoplakia. |
|  | Sui hua xiang ke ke | Herb | *Teucrium japonicum* Willd. | Whole plant | Decoction | Mountain slopes | Treat exogenous wind-cold. |
| [Feng huang cai](https://www.iplant.cn/info/%E5%87%A4%E5%87%B0%E8%8D%89) | Tie zhou cao | Semi-shrub | *Teucrium quadrifarium* Buch.-Ham. | Whole plant, Roots, Leaves | Decoction, Infuse in wine, Grind into power | On the shady slopes of mountains, Bushes | Treat edema, the roots can cure abdominal distension, the leaves can stop bleeding and treat wounds caused by knives and guns. |
| [Chong tian pao](https://www.iplant.cn/info/%E5%86%B2%E5%A4%A9%E6%B3%A1) | Xue jian chou | Herb | *Teucrium viscidum* Blume | Whole plant | Make paste by decocting, Decoction, Apply by pounding | The edge of the forest, Wasteland | Used for rheumatic arthritis and traumatic injuries. |
